# Understanding clinician attitudes towards implementation of guided self-help cognitive behaviour therapy for those who hear distressing voices: using factor analysis to test normalisation process theory

**DOI:** 10.1186/s12913-017-2449-z

**Published:** 2017-07-24

**Authors:** Cassie M. Hazell, Clara Strauss, Mark Hayward, Kate Cavanagh

**Affiliations:** 1 School of Psychology, University of Sussex, Falmer, Brighton, BN1 9QJ UK; 2 R&D Department, Sussex Partnership NHS Foundation Trust, Sussex Education Centre, Hove, BN3 7HZ UK

**Keywords:** Normalisation process theory, Implementation, Questionnaire, CBTp, CBTv, Psychosis, Hearing voices, Auditory hallucinations, Self-help, IAPT

## Abstract

**Background:**

The Normalisation Process Theory (NPT) has been used to understand the implementation of physical health care interventions. The current study aims to apply the NPT model to a secondary mental health context, and test the model using exploratory factor analysis. This study will consider the implementation of a brief cognitive behaviour therapy for psychosis (CBTp) intervention.

**Methods:**

Mental health clinicians were asked to complete a NPT-based questionnaire on the implementation of a brief CBTp intervention. All clinicians had experience of either working with the target client group or were able to deliver psychological therapies. In total, 201 clinicians completed the questionnaire.

**Results:**

The results of the exploratory factor analysis found partial support for the NPT model, as three of the NPT factors were extracted: (1) coherence, (2) cognitive participation, and (3) reflexive monitoring. We did not find support for the fourth NPT factor (collective action). All scales showed strong internal consistency. Secondary analysis of these factors showed clinicians to generally support the implementation of the brief CBTp intervention.

**Conclusions:**

This study provides strong evidence for the validity of the three NPT factors extracted. Further research is needed to determine whether participants’ level of seniority moderates factor extraction, whether this factor structure can be generalised to other healthcare settings, and whether pre-implementation attitudes predict actual implementation outcomes.

**Electronic supplementary material:**

The online version of this article (doi:10.1186/s12913-017-2449-z) contains supplementary material, which is available to authorized users.

## Background

Cognitive behaviour therapy (CBT), delivered over a minimum of 16 sessions, is the only individual psychological therapy recommended for the treatment of psychosis in a number of countries [1–4]. However, access to CBT for psychosis (CBTp) is poor, with recent figures from the UK suggesting only 10% of people with a psychosis diagnosis are offered CBTp [5]. The poor implementation of CBTp is not just limited to the UK, but is an international problem that is reported in the United States [6–9], Canada [10], and Australia [11]. The CBTp access rates are not available for many countries, but it is likely that if these more affluent countries are not able to facilitate access, then countries with less economic resource would also experiencing implementation challenges.

One of the most commonly cited reasons for the poor access to CBTp is a lack of resources, including lack of protected time and staff shortages [12]. One possible approach to increase access to CBTp is by developing interventions that can be delivered using comparatively less resources. A recent meta-analysis found briefer forms of CBTp (i.e. fewer than the recommended 16 therapy sessions) led to a significant reduction in psychosis symptoms compared to control conditions [13] These brief CBTp interventions typically targeted a specific symptom associated with psychosis (e.g. delusions or voices). Consequently, there is potential for brief forms of symptom-specific CBTp to be offered in the first instance. Drawing on a stepped care approach [14], more resource intensive forms of CBTp could then be delivered only to those still in need. However, based on the broader CBTp literature, we know demonstrating effectiveness does not necessarily lead to widespread implementation. For example, a recent audit of CBTp implementation within a NHS healthcare trust found that only 6.9% of people with psychosis were offered CBT, despite NICE [2] recommending that everyone with psychosis should be offered CBTp [15]. Therefore, in addition to investigating the effects of brief CBTp, we need to consider the potential challenges and facilitators to implementing this novel intervention.

This problem of implementation does not just apply to CBTp. It is common for healthcare services to experience delays in the process of implementing new treatments more broadly [16]. The difficulties associated with implementation has led to the development of numerous theoretical models that aim to understand and simplify this process [17]. A review by Tabak, Khoong, Chambers and Brownson [18] identified 12 separate models of implementation; however only two of these (Conceptual Model of Implementation Research, [19]; Normalisation Process Theory, [20]) consider implementation at multiple levels, including the individual and system levels.

The Conceptual Model of Implementation Research [19] synthesises previous theories of implementation to create a model that suggests different ways that implementation can be conceptualised (i.e. systems environment, organisational, learning, supervision, individual providers), and measured (i.e. feasibility, fidelity, penetration, acceptability, sustainability, uptake, and costs). The main purpose of this model is to explain the different outcomes that can be used to assess implementation, and the relationship between these outcomes [21]. Although this model is useful, it is not appropriate to be used within the present study, as its purpose is not in line with our study aims. This study aims to explore the barriers and facilitators to implementing a brief CBTp intervention prospectively; whereas the Conceptual Model of Implementation Research considers implementation retrospectively, and does not include a framework for exploring what these barriers and facilitators could be.

Conversely, the flexibility of the Normalisation Process Theory (NPT; [20]) means it can be appropriately applied to the present research study. NPT provides a theoretical framework to guide the implementation process. The theory specifies four factors that may enhance the likelihood of successfully implementing a new idea into an existing service: (1) coherence: the attitude of staff towards the new idea, (2) cognitive participation: the willingness of staff to be involved in implementation, (3) collective action: service level pragmatics involved in implementation, and (4) reflexivity: how the implementation process should be evaluated. This model can be used to consider the implementation of a brief CBTp intervention prospectively, and the NPT factors provide a theoretical basis from which barriers and facilitators can be explored.

NPT [20] has been applied to many different healthcare interventions and contexts, including physical health, service infrastructure, and mental health [22]. Looking specifically at the mental health related research, NPT [20] has been used to explore the implementation of stepped care [23], depression interventions [24] and collaborative care [25], primary mental health care [26], bipolar treatment guidelines [27], and problem-solving therapies [28]. Some of these studies applied the NPT model [20] retrospectively as a means of reviewing a previous implementation process e.g. [26]; and some utilised NPT [20] prospectively to develop an implementation plan e.g. [25]. All of these studies used qualitative research methods to understand implementation within the NPT framework, and all concluded that NPT was a useful and comprehensive model to guide the implementation process in mental health service settings. There are currently no studies however that have tested the validity of the NPT model using a quantitative design in a mental health context.

A NPT questionnaire has recently been developed (NoMAD; [29]). The psychometric properties of this measure are currently under assessment [30]. Similar to the Conceptual Model of Implementation Research [19], the items are phrased to look at implementation retrospectively. Furthermore, while the NoMAD measure is suitably vague to enable its use across multiple settings, this does limit its practical use in certain contexts. For example, one item on the NoMAD asks whether ‘sufficient resources are available to support the intervention’. In the context of our brief CBTp intervention, resources can be taken to mean the number of clinicians, clinician’s time, training, or information [12]. Consequently, we have developed our own questionnaire measure based on the NPT model [20] that has been specifically developed to investigate the prospective implementation of a brief CBTp intervention.

We plan to implement a brief CBTp intervention for distressing voices (CBTv) into National Health Service (NHS) mental health services in the UK [31]. The intervention is designed for adults who are distressed by hearing voices and who are currently receiving mental health care in an NHS service. Services would most typically be either secondary care community teams or early intervention for psychosis services. Accredited therapists generally have a positive attitude towards, and frequently use, self-help materials in their clinical work [32]. In contrast, mental health nurses have reported feeling sceptical about the value, and even the appropriateness, of talking to people about their voice hearing experiences [33]. These differing attitudes towards these aspects of guided self-help CBTv, suggests that mental health practitioners as a workforce may not be a homogeneous population. This has implications for our study as the findings from our NPT questionnaire could be moderated by the sample characteristics.

In light of this research, our study aims to: (1) test the validity of the four factor NPT model within our questionnaire using factor analysis; and based on the established factor structure, (2) identify mental health practitioner views on the implementation of guided self-help CBTv, and what sample characteristics may moderate these views. To meet our second study aim we will explore the following research questions: (a) Do attitudes differ between those who do and do not have accreditation to deliver therapy? (b) Do attitudes differ depending on the participants’ level of experience working with people who hear voices?

## Methods

### Design

This was a cross-sectional study using self-report questionnaires to seek clinicians’ views about guided self-help CBTv.

### Ethics, consent and permissions

The study received ethical approval from the Sciences and Technology C-REC at the University of Sussex, UK (Reference: ER/CH283/4). NHS Research Governance approval was granted by the Sussex Partnership NHS Foundation Trust. Participants gave informed consent for their participation in this study.

### Participants

The study inclusion criteria required that participants were clinicians working in an NHS mental health service, and also had experience of delivering psychological interventions and/or experience of working with clients who hear voices. To elaborate upon the terminology used in this paper, (1) Psychological Wellbeing Practitioners (PWPs) refers to clinicians with a year-long training in guiding CBT-based approaches for anxiety and depressive disorders, but they are not trained to the level of a CBT therapist; (2) Psychological Therapists refer to clinicians with a formal psychological therapy training which would include CBT therapists, Clinical Psychologists, and Counselling Psychologists, they may or may not have experience of working with people who hear voices; (3) Mental Health Professionals refers to clinicians with a core profession (e.g. nurses, psychiatrists, and occupational therapists) but without a formal psychological therapy training – they would be expected to have experience working with people who hear voices; and (4) Support Workers refers to clinicians who have no formal mental health qualification and no formal therapy training, but would be expected to have worked with people who hear voices in a support capacity. There were no exclusion criterion.

A total of 201 mental health clinicians, working in an NHS mental health trust in the South of England, participated in the survey. See Table 1 for information on the participant characteristics.

**Table 1:** Participant Characteristics: *N* = 201

Age (years)*M(SD)*	42.68 (10.58)
Gender %	
Male	25.9
Female	73.6
Prefer Not to Say	0.5
Team %	
Primary Care	5.0
Secondary Care	86.5
Early Intervention in Psychosis	8.5
Profession %	
Psychological Therapist	27.9
Psychological Wellbeing Practitioner (PWP)	1.5
Mental Health Professional	56.6
Support Worker	14.0
Duration in Profession (years) *M(SD)*	13.71 (10.40)

### Materials

We developed a questionnaire to assess clinicians’ attitudes towards guided self-help CBTv in relation to each of the four NPT factors [20]. The NPT questionnaire was developed in three phases, in line with the questionnaire development guidelines by Finch et al. [34]: (1) use of empirical knowledge, (2) use of expert opinion, and (3) use of theory.

Phase One (Use of Empirical Knowledge): Items were informed by the findings of a meta-analysis of briefer forms of CBTp (i.e. <16 sessions) [13]. Notably, the meta-analysis supported continued research on brief CBTp, as well as the adoption of the symptom-specific approach. Items were also informed by the implementation literature for CBTp more broadly, and included all of the known barriers to CBTp e.g. lack of time, high workloads, inadequate training [35, 36].

Phase Two (Use of Expert Opinion): Members of the research team included two experts in CBTp and an expert in self-help CBT approaches. They drew on their expertise to generate items for the self-report questionnaire.

Phase Three (Use of Theory): As mentioned previously, this questionnaire was based upon the NPT model [20]. Items were developed to address each of the NPT factors: (1) coherence (12 items) e.g. “I would be happy to refer a client who hears distressing voices to receive guided self-help CBT for distressing voices”; (2) collective action (9 items) e.g. “The resources needed to trial guided self-help CBT for distressing voices are available”, (3) cognitive participation (10 items) e.g. “I would like to be involved in research that is trialling guided self-help CBT for distressing voices”; and (4) reflexive monitoring (9 items) e.g. “Measures of symptom severity e.g. psychosis measures, are a good way to evaluate the effectiveness of guided self-help CBT for distressing voices”.

The resultant items were discussed amongst the research team, editing and removing items as required until consensus amongst the team was reached. The subsequent questionnaire included 40 items (see Additional file 1). In line with questionnaire design best practice [37], eight items were negatively worded. The questionnaire also included four free text boxes, to give participants the opportunity to elaborate on their responses. The questionnaire used a 7 point Likert scale, from strongly agree to strongly disagree. A score of 7 reflects a strong negative attitude, 4 reflects a neutral response, and 1 reflects a strong positive attitude.

### Procedure

Mental health clinicians meeting the inclusion criteria were invited to complete the questionnaire either online (using Bristol Online Survey) or on a hard copy. Participants were informed that consent was to be assumed if they returned their completed questionnaire. All of the items and free text boxes were optional questions so that participants could decline to answer any of the items. The questionnaires were completed anonymously to allow clinicians the freedom to express negative views.

### Planned analysis

Participants’ responses to the NPT questionnaire were transferred to SPSS version 22. All items that were negatively worded were reverse-scored at this point. Items were initially screened using the criteria suggested by Field [38] to determine whether they met criteria for factor analysis: the standard deviations, skew and kurtosis of the items were screened to ensure that none were outliers or significantly non-normal. The inter-item correlations and multicollinearity statistics were also checked to ensure that items were related and additive.

To address our first aim, an exploratory factor analysis (EFA) with a principal axis factoring method was used to establish a factor structure. An oblique rotation (specifically Direct Oblimin) was used as factors are expected to be related; the number of iterations for the rotation was set to 50. Exploratory factor analysis was chosen in favour of confirmatory factor analysis (CFA) as this is the first empirical assessment of the NPT model, and EFA does not make any assumptions about the model that will emerge. EFA will therefore provide a more rigorous test than CFA of the NPT model. That is, EFA will allow the model with the best fit to the data to emerge without any theoretical constraints. If the resultant factor structure supports the NPT model this would provide strong evidence for the model (as any model was free to emerge). Factor extraction was initially based upon eigenvalues reaching greater than one, as per the recommendations of Kaiser [39]. This criteria for factor extraction can only be used if Kaiser’s [39] criteria are met (i.e. there are fewer than 30 items and all communalities after extraction are greater than 0.7). The second of Kaiser’s [39] criterion does not apply to this analysis as the sample size is less that 250 (*n* = 201). If Kaiser’s [39] criteria was not met, then, as the sample contains more than 200 participants, factor extraction can be conducted using a Scree plot. Factor loadings that were less than .4 were suppressed. Where items loaded onto more than one factor at .4 or greater, the item was assigned to the factor with which it made most conceptual sense. The reliability of the factors was assessed using Cronbach’s alpha; the interpretation of scale reliability follows the guidance suggested by Tavakol and Dennick [40]. Subsequently, a scale was interpreted as reliable if the value of Cronbach’s alpha was between .70 and.95.

In order to address the second aim, mixed-design ANOVAs were planned to explore group-level differences. The following research questions were explored: (a) Do attitudes differ between those who do and do not have accreditation to deliver therapy? (b) Do attitudes differ depending on the participants’ level of experience working with people who hear voices? Post hoc tests with Bonferroni corrections were used where significant main effects were found. The post hoc test used for between group analyses was chosen in line with recommendation from Field [38] based on whether group sizes and variances were equal or not.

## Results

### Aim 1: Establishing the factor structure

Items were initially removed if more than 20% of the inter-item correlations were non-significant at the *p* < .05 level; this resulted in 7 items being removed (J1, K1, N1, P1, A2, H2 and K2). Secondly, items were removed if either the item standard deviation was greater than 2.5, or both skew and kurtosis Z scores were significantly different from normal at the *p* < .001 level; 7 more items were removed (C1, M1, D2, E2, J2, M2 and O2). None of the inter-item correlations suggested multicollinearity (all *r*s < .80). The remaining 26 items were included in the subsequent principal axis factor analysis.

Kaiser’s [39] criteria were not met, as communalities were below 0.7 after extraction (lowest 0.26); therefore the factor structure extracted based on the eigenvalues is not reliable. Consequently we used a Scree plot to determine the number of factors to be extracted. The inflexion on the Scree plot suggested a three factor solution. The EFA was re-run forcing three factors to be extracted. Six more items were removed at this stage, as factor loadings were below .4 (B2, C2, G2, I2, L2 and P2); resulting in 20 items being included in the final EFA. The present sample size was deemed ‘meritorious’ (KMO = .88; *χ* ^2^(190) = 1940.18, *p* < .001) [41].

The EFA required five iterations to converge. The three factor structure explained 50.14% of the total variance. Table 2 shows the results of the final factor structure. None of the items cross loaded. The first factor includes 8 items that all relate to attitudes towards the concept of guided self-help CBTv and conceptually fits with the NPT construct of ‘coherence’. The 6 items within the second factor all enquire about willingness to be involved in different aspects of the intervention and conceptually fits with the NPT construct of ‘cognitive participation’. The final factor has 6 items that ask about the different ways that the intervention could be evaluated to examine if it has been effective. This factor conceptually fits with the NPT construct of ‘reflexive monitoring’. It is noteworthy that three of the four proposed NPT factors emerged from the EFA, with one of the NPT factors (‘collective action’) not being represented.

**Table 2:** Final factor structure of staff questionnaire on guided self-help CBT invention for Voices

	M	SD	*α*	1	2	3
1 Coherence	2.63	0.92	.89			
Guided self-help CBT for distressing voices is an appropriate treatment option				0.86		
I would be happy to refer a client who hears distressing voices to receive guided self-help CBT				0.80		
I would be willing to refer a client who hears distressing voices to receive guided self-help CBT as part of a research project				0.69		
Guided self-help CBT for distressing voices would be effective for those with long standing symptoms				0.68		
Guided self-help CBT for those who hear distressing voices would be unsafe^a^				0.67		
It is a waste of resources to trial guided self-help CBT for those who hear distressing voices^a^				0.64		
Guided self-help CBT for those who hear distressing voices will be very effective				0.61		
People who hear distressing voices would not be able to engage in guided self-help CBT^a^				0.61		
2 Cognitive participation	2.76	1.11	.86			
I would be willing to have training to be able to deliver guided self-help CBT for distressing voices					0.86	
I would be willing to deliver guided self-help CBT for distressing voices as part of my job					0.79	
It would be possible to find the time to attend two day training course on how to deliver guided self-help CBT for distressing voices					0.66	
I would be willing to be involved in the development of guided self-help CBT for those with distressing voices					0.62	
I would be willing to be involved in research that is trialling guided self-help CBT for distressing voices					0.57	
I would not be prepared to receive training to deliver guided self-help CBT for distressing voices^a^					0.55	
3 Reflexive Monitoring	2.28	0.74	.79			
Research is a good method of testing a new intervention						0.72
Following clients up after a period of several months to administer clinical measures is a good way to evaluate the effectiveness of guided self-help CBT for distressing voices						0.68
Measures of the distress experience from hearing voices is a good way to evaluate the effectiveness of guided self-help CBT for distressing voices						0.68
Measures of symptom severity e.g. psychosis measures, are a good way to evaluate the effectiveness of guided self-help CBT for distressing voices						0.60
Randomised controlled trials e.g. comparing the treatment to a control group, is a good way to evaluate the effectiveness of guided self-help CBT for distressing voices						0.52
Measures of other clinical symptoms e.g. anxiety and depression, are a good way to evaluate the effectiveness of guided self-help CBT for distressing voices						0.49

α = Cronbach’s alpha; ^a^Items have been reverse scored; Scale scores: from 1 (positive attitude) to 7 (negative attitude)

All of the scales had good internal consistency (lowest Cronbach’s α = .79; see Table 2). The reliability of all scales could not be improved by removing any of the items. Furthermore all of the items correlated moderately well with the associated scale total (Idea: lowest *r* = .57; Involvement: lowest *r* = .54; Evaluation: lowest *r* = .52).

### Aim 2: Clinicians’ attitudes towards guided self-help CBT for voices

The attitude scores, for all of the factors, were significantly lower than 4 (representing a neutral attitude) (Coherence: *t*(200) = −21.03, *p* < .001; Cognitive Participation: *t*(199) = −15.76, *p* < .001; Reflexive Monitoring: *t*(200) = −33.04, *p* < .001). This finding suggests that attitudes were generally positive in relation to each of the factors; see Tables 2 and 3 for the descriptive statistics.

**Table 3:** Descriptive statistics for all factors across participant characteristics

		Coherence			Cognitive Participation			Reflexive Monitoring		
	*n*	M	SD	*d*	M	SD	*d*	M	SD	*d*
Accredited Therapist				0.47			0.31			0.03
Yes	59	2.94	0.90		3.00	1.20		2.30	0.80	
No	140	2.52	0.90		2.66	1.07		2.28	0.71	
Experience working with people who hear voices				−0.21			0			−0.12
A lot to moderate	166	2.61	0.90		2.76	1.12		2.27	0.71	
Little to none	34	2.80	1.00		2.76	1.13		2.36	0.87	

*d* = Cohen’s *d*

Mauchly’s test indicated that the assumption of sphericity had been violated (*W* = .95; *χ* ^2^(2) = 10.35, *p* = .006); therefore a Greenhouse-Geisser correction was used for all ANOVAs involving repeated measures (as *ε* > .75).

### Are attitudes different across the factors between those who do and do not have accreditation to deliver therapy?

There was a significant main effect of therapist qualification on the ratings given across factors (*F*(1, 197) = 5.01, *p* = .03). Those qualified to deliver psychological therapy (*EMM* = 2.75; *SE* = 0.10) gave generally higher (less favourable) ratings than non-therapists (*EMM* = 2.49; *SE* = 0.06). There was a significant interaction between the factors and whether participants were qualified to deliver therapy or not (*F*(1.89, 371.27) = 3.79, *p* = .03). The only factor where therapists and non-therapists differed significantly was on the Coherence factor, where therapists gave significantly less favourable ratings compared to non-therapists (Therapists: *M* = 2.94, 95% CI [2.71, 3.17]; Non-therapists: *M* = 2.52, 95% CI [2.37, 2.67]), representing a medium sized effect (Cohen’s *d* = 0.47) [42].

### Do attitudes differ across the factors depending on the level of experience the participant has working with people who hear voices?

There was no significant main effect of experience working with people who hear voices on subscale scores (*F*(1, 198) = 0.47, *p* = .49). Also the interaction between the factors and level of experience with clients who hear voices was non-significant (*F*(1.90, 377.01) = .52, *p* = .58). These findings suggest that ratings on subscales were not affected by participant’s level of experience of working with people who hear voices.

## Discussion

The first aim of our study was to test the proposed factor structure of Normalisation Process Theory (NPT) [20] using exploratory factor analysis. We found partial support for the NPT model as the three factors extracted were akin to three of the NPT factors: (1) coherence, (2) cognitive participation, and (3) reflexive monitoring. This study seems to be the first test of the NPT model using factor analysis, although the findings of the NoMAD factor analysis are imminent [30]. Our findings suggest that coherence, cognitive participation, and reflexive monitoring are important facets of implementation that should be considered prior to the dissemination of brief CBTp interventions. This result is particularly compelling as the use of EFA meant that any factor structure could have emerged. It is possible that these three factors would also emerge as important when understanding the implementation of healthcare interventions more generally. However, as our study asked participants about a specific intervention (guided self-help CBTv) further research using factor analysis is required to determine the generalizability of the NPT model.

We failed to find support for the fourth NPT factor, collective action, which speaks to the feasibility of implementing the new intervention into the existing service. This study recruited clinicians currently working in mental health services, rather than staff in more senior positions – such as service managers. Clinicians generally do not have the power or responsibility to make service-level decisions. As the collective action factor describes a facet of implementation that occurs at the service-level, this factor could be argued as inconsequential to our sample, and therefore explain why this factor did not emerge in our analysis. If participants in these more senior positions had been recruited to the study, it is possible that we may have found support for the collective action factor.

A kin to our study, most NPT studies in mental health settings recruited practitioners [24, 25, 28]. However the NPT studies by Gask et al. [26] and Franx et al. [23] did include service leads and managers to investigate the implementation of mental health care, and stepped care, into primary care services respectively. Both of these studies found qualitative support for the Collective Action factor, as having coherent and consistent leadership across the services was associated with successful implementation. However, neither study explored the moderating effect of profession, nor did they validate the NPT model quantitatively. Consequently we suggest that future tests of the NPT model should include participants of varying levels of seniority. These studies would benefit from the use of quantitative, moderation analysis to explore the effects of seniority, and address this limitation of our study and previous research.

The second aim of our study was to examine clinicians’ attitudes towards guided self-help CBTv, and whether these differed as a function of therapy training (therapist versus non-therapist) and experience working with clients who hear voices. We found that clinicians’ attitudes were favourable across all three factors (all *M*s. < 3; see Table 2). With respect to each of the factors extracted, these ratings can be interpreted to mean clinicians are, on average, supportive of the concept of guided self-help CBTv (coherence), are willing to be involved in the implementation (cognitive participation), and agree with the proposed means of evaluating the implementation (reflexive monitoring). These are encouraging findings as they suggest that most clinicians working in NHS mental health services in the UK have a positive attitude about guided self-help CBTv and would be willing to support its implementation and evaluation. This suggests that clinicians’ attitudes and willingness to be involved would not be barriers to implementation of guided self-help CBTv in the NHS.

Only therapist training significantly moderated the clinicians’ attitude, with qualified therapists reporting significantly less favourable attitudes on the coherence subscale compared to non-therapists. In practical terms however, this difference may be negligible as the mean difference between these groups was 0.42 on the seven point Likert scale. In addition, in both cases, therapists and non-therapists had mean ratings that were in the favourable range (<3) suggesting that whilst therapists were somewhat more sceptical, they were still, on average, positive in their attitudes towards to the intervention. However, there is some evidence to suggest attitudes towards psychological therapy can vary as a function of the clinicians’ profession and training. The majority of the literature suggests therapists view psychosocial interventions for psychosis with a greater optimism compared to other mental health professionals [42]. However there is some evidence that concurs with our findings, as therapists seem to be more pessimistic than mental health nurses about their ability to ‘treat’ psychosis [43]. Therapists also report that delivering brief CBTp interventions can be problematic owing to the limited number of sessions involved, and the complex nature of many patients’ presenting problems [44]. Whether the therapists’ reservations are realised in practice requires further research.

Overall, it seems clinicians show support for implementing guided self-help CBTv, which is encouraging. This finding contrasts with previous research that suggests mental health clinicians may not be supportive of interventions that invite people to talk about the voices they experience [33]. Perhaps the recent growth of emancipatory approaches to voices, such as the Hearing Voices Movement, has helped to demonstrate the therapeutic value of openly discussing voices [45]. Whether clinicians’ positive pre-implementation attitudes will aid the actual implementation process remains to be seen. There is evidence to suggest that negative clinician attitudes are associated with poorer intervention outcomes [46]. The favourable attitudes of clinicians in the present study are therefore welcome as this will help to create the optimal service environment, from which we can explore the effectiveness of this intervention.

### Limitations

It is possible that the reason we did not find support for the fourth NPT factor is because the items developed to target this factor were poor representations of the collective action factor. That is to say, our items may not have sufficiently examined implementation at the service-level. Before accepting this as a study limitation, it is important to first explore whether the sample characteristics may have contributed to the factor structure extracted. As mentioned previously, future studies should aim to include participants in more senior-level positions to see whether this causes the emergence of the collective action factor.

Our questionnaire was designed to investigate implementation prospectively. Our findings are therefore indicative of mental health practitioners anticipated barrier and facilitators to implementation – our study cannot determine whether this will translate into practice. This limitation highlights the benefits of longitudinal implementation research. Using this research design will help to determine whether positive pre-implementation attitudes translate into a successful initial implementation, and the sustained employment of the intervention. To our knowledge, there are currently no quantitative studies that have looked at the NPT’s model ability to predict implementation outcomes; however this is one of the study aims of the NoMAD psychometric assessment [30] which is currently underway.

The measure we have developed appears to have factorial validity. However, we did not assess other forms of validity such as construct and divergent validity. This will be the focus of future research evaluating the psychometric properties of the measure. Furthermore, factors were extracted using a combination of Kaiser’s [39] criteria and the Scree Plot. Although this method of factor extraction is arguably the most widely used and well-established in scale construction, other methods such as parallel analysis are gaining support [47].

### Research implications

Our study has identified a number of areas for future research. For example, future studies assessing the validity of the NPT model [20] should aim to recruit participants of varying levels of seniority, and determine whether pre-implementation attitudes correspond to the subsequent ease of implementation. As our study seems to be the first to use factor analysis to test the NPT model, more factor analysis studies are needed to see whether the NPT factor structure can be generalised to the implementation of other interventions in both mental health and physical health contexts.

## Conclusions

The present study used exploratory factor analysis to test the application of the NPT model [20] in a mental health setting. We found support for three of the NPT factors when considering the implementation of a brief CBTp intervention. The fourth factor, collective action, was not extracted. Clinicians generally supported the implementation of the intervention. The feedback from clinicians can be used to inform both research and intervention protocols. In time, we will be able to assess whether clinicians’ pre-implementation attitudes impact upon the subsequent implementation process.

## Additional file

Additional file 1: Supplementary file - copy of questionnaire. (DOCX 38 kb)

## Abbreviations

CBT: Cognitive behaviour therapy
CBTp: Cognitive behaviour therapy for psychosis
CBTv: Cognitive behaviour therapy for voices
EFA: Exploratory factor analysis
NHS: National Health Service
NPT: Normalisation process theory
UK: United Kingdom
US: United States
