# Supplementary material for: Understanding clinician attitudes towards implementation of guided self-help cognitive behaviour therapy for those who hear distressing voices: using factor analysis to test normalisation process theory
Source: BMC Health Serv Res. 2017 Jul 24;17:507. doi: 10.1186/s12913-017-2449-z (PMC5525252; doi:10.1186/s12913-017-2449-z)
Supplement: Additional file 1: — Supplementary file - copy of questionnaire. (DOCX 38 kb) [file 12913_2017_2449_MOESM1_ESM.docx]

**Staff Survey:**

Attitudes Towards Guided Self-Help CBT for Distressing Voices

This questionnaire is free to use for research purposes and in non-profit making organisations but please let us know if you plan to use it by emailing Clara Strauss at [c.y.strauss@sussex.ac.uk](mailto:c.y.strauss@sussex.ac.uk).

If using this questionnaire in your research please cite the published paper as Hazell, C. M., Strauss, C., Hayward, M., & Cavanagh, K. (in press). Understanding clinician attitudes towards implementation of guided self-help cognitive behaviour therapy for those who hear distressing voices: Using factor analysis to test Normalisation Process Theory. *BMC Health Services Research*

**About You:**

**1. Age:**

……………………………………………………

**2. Gender:**

Male ☐

Female ☐

Prefer not to say ☐

**3. What team are you part of within mental health services?**

Primary care ☐

Assessment and treatment ☐

Recovery and wellbeing ☐

Inpatient ☐

Early intervention ☐

Assertive outreach ☐

Other *(please specify below):* ☐

…………………………………………………………………………………………

**4. What is your profession?**

Psychological wellbeing practitioner (Step 2 practitioner) ☐

Nursing ☐

Social work ☐

Occupational therapy ☐

Support worker ☐

Clinical/counselling psychologist ☐

CBT therapist ☐

Counsellor or other psychotherapist ☐

Other *(please specify below):* ☐

…………………………………………………………………………………………

**5. How long have you been in this profession?** *(Please give your answer in years)*

……………………………………………………

**6. Within your work in mental health services, how much experience do you have working with people who are distressed by hearing voices?**

A lot (worked with 10+ people distressed by hearing voices) ☐

Some (worked with 5-9 people distressed by hearing voices) ☐

A little (worked with 1-4 people distressed by hearing voices) ☐

None (no experience with this group) ☐

**7. How much cognitive behavioural therapy (CBT) training have you received?**

Qualified CBT therapist or equivalent ☐

Qualified psychological wellbeing practitioner or equivalent ☐

In training as a CBT therapist or equivalent ☐

In training as a psychological wellbeing practitioner or equivalent ☐

10 or more days of CBT training ☐

5 to 9 days of CBT training ☐

1 to 4 days of CBT training ☐

No formal CBT training but use ideas in my work ☐

No formal CBT training and do not use CBT ideas in my work ☐

Below are a range of statements aimed to understand your opinion on the concept and implementation of guided self-help CBT for distressing voices.

Please read each statement carefully and give your honest answer. Please try to answer all the questions below.

**Section One:**

8. Select the answer that best fits your opinion for each statement.

| 1 | 2 | 3 | 4 | 5 | 6 | 7 |
| --- | --- | --- | --- | --- | --- | --- |
| Strongly Agree | Agree | Somewhat Agree | Neither Agree nor Disagree | Somewhat Disagree | Disagree | Strongly Disagree |

| A | Randomized controlled trials e.g. comparing the treatment to a control group, is a good way to evaluate the effectiveness of guided self-help CBT for distressing voices | 1 | 2 | 3 | 4 | 5 | 6 | 7 |
| --- | --- | --- | --- | --- | --- | --- | --- | --- |
| B | Guided self help CBT for distressing voices is an appropriate treatment option | 1 | 2 | 3 | 4 | 5 | 6 | 7 |
| C | Qualitative measures e.g. interviews after the intervention, are a good way to evaluate the effectiveness of guided self help CBT for distressing voices | 1 | 2 | 3 | 4 | 5 | 6 | 7 |
| D | Guided self help CBT for distressing voices would be effective for those with long standing symptoms | 1 | 2 | 3 | 4 | 5 | 6 | 7 |
| E | I would be willing to be involved in the development of guided self help CBT for those with distressing voices | 1 | 2 | 3 | 4 | 5 | 6 | 7 |
| F | I would be willing to refer a client who hears distressing voices to receive guided self help CBT as part of a research project | 1 | 2 | 3 | 4 | 5 | 6 | 7 |
| G | I would be happy to refer a client who hears distressing voices to receive guided self help CBT | 1 | 2 | 3 | 4 | 5 | 6 | 7 |
| H | It is a waste of resources to trial guided self help CBT for those who hear distressing voices | 1 | 2 | 3 | 4 | 5 | 6 | 7 |
| I | I would be willing to be involved in research that is trialing guided self help CBT for distressing voices | 1 | 2 | 3 | 4 | 5 | 6 | 7 |
| J | Attending a separate supervision for those who deliver guided self help CBT for stressing voices would not be practical for me | 1 | 2 | 3 | 4 | 5 | 6 | 7 |
| K | Self help materials e.g. books, would be effective for those that hear distressing voices | 1 | 2 | 3 | 4 | 5 | 6 | 7 |
| L | People who hear distressing voices would not be able to engage in guided self help CBT | 1 | 2 | 3 | 4 | 5 | 6 | 7 |
| M | It is not possible to implement guided self help CBT, within existing mental health services | 1 | 2 | 3 | 4 | 5 | 6 | 7 |
| N | It would be possible to free up time to deliver guided self help CBT for distressing voices | 1 | 2 | 3 | 4 | 5 | 6 | 7 |
| O | It would be possible to find the time to attend a two day training course on how to deliver guided self help CBT for distressing voices | 1 | 2 | 3 | 4 | 5 | 6 | 7 |
| P | Majority of my clients who hear distressing voices would like to receive guided self help CBT | 1 | 2 | 3 | 4 | 5 | 6 | 7 |
| Q | I would not be prepared to receive training to deliver guided self help CBT for distressing voices | 1 | 2 | 3 | 4 | 5 | 6 | 7 |
| R | I would be willing to deliver guided self help CBT for distressing voices as part of my job | 1 | 2 | 3 | 4 | 5 | 6 | 7 |
| S | Measures of other clinical symptoms e.g. anxiety and depression, are a good way to evaluate the effectiveness of guided self help CBT for distressing voices | 1 | 2 | 3 | 4 | 5 | 6 | 7 |
| T | Measures of symptom severity e.g. psychosis measures, are a good way to evaluate the effectiveness of guided self help CBT for distressing voices | 1 | 2 | 3 | 4 | 5 | 6 | 7 |

**Section Two:**

9. Select the answer that best fits your opinion for each statement.

| 1 | 2 | 3 | 4 | 5 | 6 | 7 |
| --- | --- | --- | --- | --- | --- | --- |
| Strongly Agree | Agree | Somewhat Agree | Neither Agree nor Disagree | Somewhat Disagree | Disagree | Strongly Disagree |

| A | Those who hear distressing voices should be able to receive the same treatment options as those with common mental health problems | 1 | 2 | 3 | 4 | 5 | 6 | 7 |
| --- | --- | --- | --- | --- | --- | --- | --- | --- |
| B | CBT can be effective for those who hear distressing voices if it is delivered by a non-therapist e.g. psychological wellbeing practitioner | 1 | 2 | 3 | 4 | 5 | 6 | 7 |
| C | I believe my colleagues would be willing to be involved in trials for guided self-help CBT for distressing voices | 1 | 2 | 3 | 4 | 5 | 6 | 7 |
| D | It would be possible to identify clients that are suitable to receive guided self-help CBT for distressing voices | 1 | 2 | 3 | 4 | 5 | 6 | 7 |
| E | Measures of quality of life e.g. engagement in meaningful activity, are a good way to evaluate the effectiveness of guided self-help CBT for distressing voices | 1 | 2 | 3 | 4 | 5 | 6 | 7 |
| F | Research is a good method of testing a new intervention | 1 | 2 | 3 | 4 | 5 | 6 | 7 |
| G | The resources needed to trial guided self-help CBT for distressing voices are available | 1 | 2 | 3 | 4 | 5 | 6 | 7 |
| H | Having a client who hears distressing voices receiving guided self-help CBT would make my workload unmanageable | 1 | 2 | 3 | 4 | 5 | 6 | 7 |
| I | My team would be able to aid in the implementation of guided self-help CBT for distressing voices | 1 | 2 | 3 | 4 | 5 | 6 | 7 |
| J | I would be willing to attend a course on the therapeutic principles of guided self-help CBT for those who hear distressing voices | 1 | 2 | 3 | 4 | 5 | 6 | 7 |
| K | Measures of acceptability e.g. client satisfaction, is a good way to evaluate the effectiveness of guided self-help CBT for distressing voices | 1 | 2 | 3 | 4 | 5 | 6 | 7 |
| L | Being trained to deliver guided self-help CBT for distressing voices would make my job harder | 1 | 2 | 3 | 4 | 5 | 6 | 7 |
| M | Guided self-help CBT for distressing voices sounds like a good idea | 1 | 2 | 3 | 4 | 5 | 6 | 7 |
| N | Guided self-help CBT for those who hear distressing voices would be unsafe | 1 | 2 | 3 | 4 | 5 | 6 | 7 |
| O | Symptom specific treatment, like guided self-help CBT for distressing voices, are a good approach to treatment | 1 | 2 | 3 | 4 | 5 | 6 | 7 |
| P | Having 6-8 sessions of guided self-help CBT for those with distressing voices would be feasible to implement as a treatment option | 1 | 2 | 3 | 4 | 5 | 6 | 7 |
| Q | Following clients up after a period of several months to administer clinical measures is a good way to evaluate the effectiveness of guided self-help CBT for distressing voices | 1 | 2 | 3 | 4 | 5 | 6 | 7 |
| R | I would be willing to have training to be able to deliver guided self-help CBT for distressing voices | 1 | 2 | 3 | 4 | 5 | 6 | 7 |
| S | Guided self-help CBT for those who hear distressing voices will be very effective | 1 | 2 | 3 | 4 | 5 | 6 | 7 |
| T | Measures of the distress experience from hearing voices is a good way to evaluate the effectiveness of guided self-help CBT for distressing voices | 1 | 2 | 3 | 4 | 5 | 6 | 7 |

**Section Three:**

10. What do you think about the idea of offering CBT for distressing voices using guided self-help? *(Optional)*

11. How willing would you be to be involved in the development of guided self-help CBT for distressing voices? *(Optional)*

12. How feasible do you think it would be to implement guided self-help CBT for distressing voices in the trust? *(Optional)*

13. How should guided self-help CBT for distressing voices be evaluated? *(Optional)*

Thank you…

Thank you for completing this survey.
